# Cortical Thickness, Surface Area and Volume Measures in Parkinson's Disease, Multiple System Atrophy and Progressive Supranuclear Palsy

**DOI:** 10.1371/journal.pone.0114167

**Published:** 2014-12-02

**Authors:** Amanda Worker, Camilla Blain, Jozef Jarosz, K. Ray Chaudhuri, Gareth J. Barker, Steven C. R. Williams, Richard Brown, P. Nigel Leigh, Andrew Simmons

**Affiliations:** 1 King's College London, Institute of Psychiatry, London, United Kingdom; 2 National Institute for Health Research Biomedical Research Centre for Mental Health at South London and Maudsley NHS Foundation Trust and Institute of Psychiatry, King's College London, London, United Kingdom; 3 National Institute for Health Research Biomedical Research Unit for Dementia at South London and Maudsley NHS Foundation Trust and Institute of Psychiatry, King's College London, London, United Kingdom; 4 King's College Hospital, London, United Kingdom; 5 Trafford Centre for Biomedical Research, Brighton and Sussex Medical School, University of Sussex, Brighton, United Kingdom; University of Manchester, United Kingdom

## Abstract

**Objective:**

Parkinson's disease (PD), Multiple System Atrophy (MSA) and Progressive Supranuclear Palsy (PSP) are neurodegenerative diseases that can be difficult to distinguish clinically. The objective of the current study was to use surface-based analysis techniques to assess cortical thickness, surface area and grey matter volume to identify unique morphological patterns of cortical atrophy in PD, MSA and PSP and to relate these patterns of change to disease duration and clinical features.

**Methods:**

High resolution 3D T1-weighted MRI volumes were acquired from 14 PD patients, 18 MSA, 14 PSP and 19 healthy control participants. Cortical thickness, surface area and volume analyses were carried out using the automated surface-based analysis package FreeSurfer (version 5.1.0). Measures of disease severity and duration were assessed for correlation with cortical morphometric changes in each clinical group.

**Results:**

Results show that in PSP, widespread cortical thinning and volume loss occurs within the frontal lobe, particularly the superior frontal gyrus. In addition, PSP patients also displayed increased surface area in the pericalcarine. In comparison, PD and MSA did not display significant changes in cortical morphology.

**Conclusion:**

These results demonstrate that patients with clinically established PSP exhibit distinct patterns of cortical atrophy, particularly affecting the frontal lobe. These results could be used in the future to develop a useful clinical application of MRI to distinguish PSP patients from PD and MSA patients.

## Introduction

Multiple System Atrophy (MSA), Progressive Supranuclear Palsy (PSP) and Parkinson's disease (PD) are pathologically distinct neurodegenerative diseases [1] that can be clinically indistinguishable, particularly in the early stages.[2],[3] The majority of MSA and PSP cases are characterised by relentless disease progression and carry a shortened life expectancy from onset of symptoms, whereas PD is not associated with a substantial reduction in life expectancy.[2],[4],[5] It is possible that these neurodegenerative diseases possess unique morphological signatures, thus detection of sensitive and specific biomarkers may prove useful in improving early diagnosis.

Neurodegenerative disorders are often associated with structural changes in the brain and magnetic resonance imaging (MRI) holds the potential to detect subtle variations in the volume and shape of subcortical regions, as well as cortical morphometric changes such as cortical volume, thickness, area and folding pattern, thus allowing the identification of objective diagnostic markers. Voxel-based morphometry (VBM) is a neuroimaging analysis technique allowing whole brain voxel-based investigation of focal changes in brain anatomy.[6] Studies exploring regional brain volume differences in Parkinsonian syndromes have revealed that the pathology of this disease is not confined to subcortical regions but may also include atrophy of the cerebral cortex, in particular the frontal cortex.[7]–[10] This frontal atrophy is especially prominent in PSP, often with a distinct pattern of superior frontal atrophy.[8] Despite several studies indicating the involvement of the cortex, the brain pathology underlying motor and non-motor symptoms in parkinsonian syndromes is incompletely defined and inferences are currently limited by the use of grey matter volume measurement.

Surface-based methods provide a sensitive tool for investigating cortical atrophy by breaking down measures of local cortical volume into separate and almost orthogonal components of thickness and surface area. While thickness measures may provide some indication of underlying neuronal loss, reduced size of neuronal cell bodies or degradation, surface area measures may reflect underlying white matter fibers [11]; with tension or shrinkage of these fibers leading to deeper sulci and extended area measures. In addition to increased pathological specificity, cortical thickness and area measures provide a direct index of cortical morphology that is less susceptible to variations in individual positioning, as the extraction of the cortex follows the grey matter surface regardless of positional variance.[12] Furthermore, cortical thickness measures have the advantage over voxel-based measures as they allow for sub-voxel precision with thickness values being assigned to individual vertices rather than voxels.[13] Studies combining measures of volume and surface-based methods measuring cortical thickness and surface area have shown that both techniques lead to similar findings but surface-based methods are able to provide more sensitivity.[14]

Cortical atrophy is frequently regionally specific in disease and the progress of atrophy can therefore reveal much about the evolution and causative factors of a disease. Few studies have used surface-based methods to assess cortical morphology in Parkinsonian syndromes, those that have, have focused on PD and identified widespread regions of cortical thinning,[15],[16] however these studies are not consistent with earlier reports of frontal atrophy. To our knowledge there have been no previous studies comparing patterns of cortical change in PD, MSA and PSP. Thus in this study we have chosen to measure cortical thickness, surface area and cortical volume using the automated surface-based analysis package FreeSurfer (Massachusetts General Hospital, Harvard Medical School; http://surfer.nmr.mgh.harvard.edu).

It is not clear which affected cortical regions differ between these three diseases and how this relates to differential clinical and cognitive symptoms. The aims of the present study are firstly to identify regions of cortical atrophy that will enable characterisation of each of the three diseases and secondly to discover whether these regions relate to clinical or cognitive symptoms of the disease. Based on the reviewed evidence, we hypothesise that along with clinical symptom severity, both the MSA and PSP groups will display marked thinning and volume loss of the cortex, particularly in frontal regions. We also hypothesise that the PD patients will show subtle cortical changes that are more widespread across the whole cortex.

## Materials and Methods

### Participants

Fourteen patients diagnosed with PSP, eighteen with MSA and fourteen with PD, according to established criteria, [17]–[19] were recruited successively from the Movement Disorders Clinic at King's College Hospital and via referrals from clinicians in south east England. Eleven probable MSA patients were categorised as MSA-P (predominant parkinsonian features) and seven as MSA-C (predominant cerebellar features); both clinical variants of MSA were included since the aim of this study was to explore the differences between cortical morphometry in the three clinical groups in comparison to healthy controls, rather than the variation between specific clinical variants. Of the fourteen PSP patients, thirteen were classified as probable and one as possible.[2] All patients with PD fulfilled criteria for definite PD [18] and sixteen MSA patients were categorised as probable, one as possible and one as definite. Nineteen healthy controls matched for age, were also recruited (spouses and friends of patients). The project was approved by research ethics committees of King's Healthcare NHS Trust and the Institute of Psychiatry and South London and Maudsley NHS Trust. Written informed consent was given by all subjects before participation in the study.

### Clinical and cognitive measures

Within 1 week of the MRI scan each participant was examined by the same clinician (CB). Disease severity was measured using Hoehn and Yahr (H&Y), Schwab and England activities of daily living (ADL) and Unified Parkinson's Disease Rating Scale Part III (UPDRS-III). Cerebellar ataxia and occulomotor dysfunction were assessed using the Parkinson's Plus Scale. Measures of global cognitive function were also taken using the Mini-Mental State Examination (MMSE) [20] and Mattis Dementia Rating Scale (DRS).[21]

### Image acquisition

T1-weighted 3-dimensional (3D) inversion recovery prepared spoiled gradient echo (SPGR) images were acquired from the whole brain on a 1.5T Signa LX NV/i system (General Electric, Milwaukee, USA), fitted with actively shielded magnetic field gradients (maximum amplitude 40 mTm ^−1^) and a standard quadrature birdcage head coil. Other parameters were TR = 18 ms; TE = 5.1 ms; TI = 450 ms; 256×152 acquisition matrix over a 240×192 mm field of view (FOV), reconstructed as a 256×256 over a 240×240 FOV, giving an in-plane pixel size of 0.9375×0.9375 mm; 124×1.5 mm thick slices. Total scan time was 8 mins 20 sec.

### Statistical Analysis

#### Clinical variables

Clinical variables were analysed using SPSS (version 20); one-way ANOVA with post-hoc Tukey tests were used to assess between-group differences in age, duration of disease, UPDRS, MMSE and DRS scores. The statistical threshold was set at p <0.05. Post-hoc t-tests were also performed to assess the difference in age between the PSP group and each other group. A Kruskall-Wallis test was run to determine if there were differences between H&Y, Schwab and England ADL, PPS Occulomotor and Cerebellar scores between clinical groups. Pairwise comparisons were performed using Dunn's (1964) procedure with a Bonferroni correction for multiple comparisons. Finally, a chi-square goodness-of-fit test was conducted on gender.

### Image Analysis

#### FreeSurfer analysis pipeline

Surface-based analysis was carried out using FreeSurfer version 5.1.0 (Massachusetts General Hospital, Harvard Medical School; http://surfer.nmr.mgh.harvard.edu). The FreeSurfer pipeline performs cortical reconstruction and subcortical volumetric segmentation including the removal of non-brain tissue (skull, eyeballs and skin), using an automated algorithm with the ability to successfully segment the whole brain without any user intervention. This process is based on a hybrid approach combining the robustness of a watershed algorithm and the accuracy of deformable surface models. Automated Talairach transformation [22] is performed, followed by segmentation of the subcortical white matter and deep grey matter structures; voxels are classified as white matter or not based on intensity and neighbour constraints. Segmentation of deep grey matter volumetric structures includes the hippocampus, amygdala, caudate, putamen and ventricles [23] – although these structures were not used in the present study. This is followed by intensity normalisation,[24] tessellation of the grey matter-white matter boundary and the grey matter-CSF boundary, automated topology correction [25],[26] and surface deformation following intensity gradients to optimally place the grey/white and grey/cerebrospinal fluid borders at the location where the greatest shift in intensity defines the transition to the other tissue class.[13] On completion of the cortical models, individual cortical folding patterns are then registered to a spherical atlas based on folding patterns, to match cortical geometry across subjects.[27] Thickness can be calculated at each location of the cortex as the distance between the white and pial surface.[13] The cerebral cortex is then parcellated into units based on gyral and sulcal structure [23] allowing local curvature and surface area measures to be computed. The current study focused on cortical thickness, surface area and cortical volume – measures that have recently been found to show significant change in Parkinsonian syndromes.[7],[8],[28]

#### Cortical thickness, surface area and volume

Entire cortex analyses were computed to explore local cortical thickness, surface area and cortical volume in PD, MSA, PSP and healthy control subjects. Statistical maps were generated using FreeSurfer's Query, Design, Estimate, Contrast (QDEC) interface. QDEC is a single-binary application included in the FreeSurfer distribution that is used to perform group averaging and inference on the cortical morphometric data produced by the FreeSurfer processing stream. First, the three diagnostic groups (PD, MSA, PSP) were compared with healthy controls (HC), followed by comparison of the three clinical groups with each other. For each hemisphere the General Linear Model (GLM) was computed vertex-by-vertex for analysis of cortical thickness, surface area and cortical volume respectively, accounting for the effects of gender and age to avoid spurious results. QDEC provides two different methods for automatically creating a design matrix: DOSS (different offset, same slope) and DODS (different offsets, different slopes). DOSS assumes different morphometric measures for all groups (different offsets) and a similar impact of disease duration in all clinical groups (same slope). DODS also assumes different offsets but a different impact of duration between the clinical groups (different slope). It is plausible that the diagnostic groups included in this study will show different cortical evolution rates, thus DODS has been employed. Cortical maps were smoothed using a 10 mm full width at half maximum Gaussian kernel and the results were visualised by overlaying significant cortical areas onto semi-inflated cortical surfaces. Multiple comparisons were corrected with a Monte Carlo Simulation using a p-value set at <0.05.

#### Correlation between cortical, clinical and cognitive measures

The association between cortical morphometric measures and clinical measures in each clinical group was also examined using QDEC. Disease duration, measures of severity as measured by H&Y, Schwab and England ADL and UPDRS-III, cerebellar ataxia and occulomotor dysfunction as measured by the Parkinson's Plus Scale and global cognitive function as measured by the DRS were included in the analyses individually as continuous covariates. Age and gender were included in these analyses as covariates of no interest.

#### Effect of cognitive impairment

Additional analyses were conducted to assess the effect of cognitive impairment on between group differences in cortical atrophy. The DRS provides the most comprehensive assessment of cognitive function of our cognitive measures, thus patients with a DRS score below the cut-off threshold (DRS score ≤ 125) were classified as cognitively impaired and excluded from this particular analysis. Of those excluded 5 were PSP patients and 2 MSA patients (n = 7).[29]

## Results

### Demographic and clinical variables

Diagnostic groups did not differ significantly in age, gender or disease duration in one-way ANOVA analyses. However, post hoc t-tests showed that the PSP group is significantly older than the MSA and HC groups. The PD group scored significantly lower on measures of disease severity; H&Y (p≤0.001), Schwab and England ADL (p≤0.001) and UPDRS III (p≤0.001), the MSA group scored significantly higher on measures of cerebellar function (p≤0.001), while the PSP group scored significantly higher on measures of occulomotor function (p≤0.001) and lower on MMSE (p≤0.001) and DRS (p≤0.001) (Table 1). A chi-square goodness-of-fit test indicated that both genders were equally represented by the participants recruited to the study (x^2^(1) = 0.015, p = 0.901).

**Table 1:** Demographic and clinical data of control subjects and patients with PD, MSA and PSP.

	Control (n = 19)	PD (n = 14)	MSA (n = 18)	PSP (n = 14)	p-value (p<0.05)
Age (SD)	63.8 (7.9)	64.6 (6.9)	62.8 (7.2)	69.4 (7.2)	0.066
Sex, M:F	10∶9	7∶7	10∶8	5∶9	0.901
Disease duration (SD)	NA	6.6 (2.0)	5.1 (2.5)	5.1 (2.1)	0.112
H&Y* median score (range)	NA	2.5 (2.0–3.0)	3.0 (2.5–5.0)	4.0 (3.0–4.0)	<0.001
Schwab and England ADL* median score (range)	NA	90% (80–100%)	70% (40–80%)	50% (20–80%)	<0.001
UPDRS III, (SD)	NA	21.8 (9.6)	36.4 (13.2)	34.6 (8.0)	<0.001
Occulomotor Score, † median (range)	NA	0.0 (0–3)	1.0 (0–5)	13.0 (7–20)	<0.001
Cerebellar Score, † median (range)	NA	0.0 (0–2)	8.5 (0–13)	2.0 (0–6)	<0.001
DRS (SD)	NA	140 (2.9)	136 (8.4)	127 (10.2)	<0.001
MMSE (SD)	NA	29.5 (1.1)	28 (2.5)	26 (2.8)	<0.001

* For patients taking levodopa drug treatment, scores given in the ‘on’ state.

† From Parkinson's Plus Scale (Cerebellar Score, maximum 24; Occulomotor Score, maximum 21).

MSA  =  multiple systems atrophy; PSP  =  progressive supranuclear palsy; PD  =  Parkinson's disease; MSA-P  =  multiple system atrophy parkinsonian variant; MSA-C  =  multiple system atrophy cerebellar variant; H&Y  =  Hoehn and Yahr; ADL  =  activities of daily living; UPDRS III  =  Unified Parkinson Disease Rating Scale-part III.

### Cortical thickness

**Figure 1:**
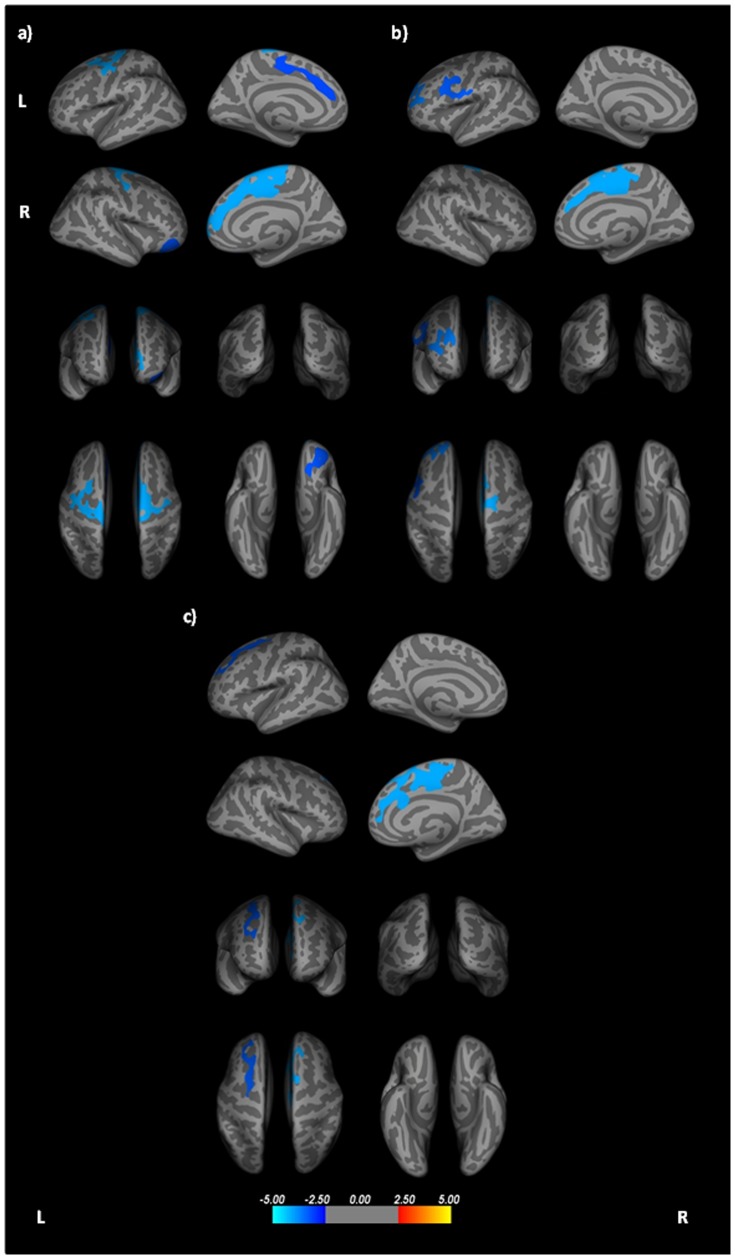
Cortical areas showing significant thinning in PSP patients compared to a) healthy controls, b) Parkinson's disease and c) Multiple System Atrophy; displayed on QDEC's semi-inflated cortical surfaces. Top row: L lateral and L medial, Second row: R lateral and R medial, Third row: anterior and posterior, Bottom row: superior and inferior views. The colour bar indicates the significance levels of the clusters. Results were obtained using Monte Carlo simulation, with a threshold of p <0.05, to provide cluster-wise correction for multiple comparisons.

PSP patients showed significant thinning of the frontal lobe, comprising the left precentral, right lateral orbitofrontal and superior frontal gyri bilaterally when compared to healthy controls (Figure 1, Table 2). When compared to the MSA group, PSP patients displayed cortical thinning of the superior frontal gyrus bilaterally (Figure 1, Table 2). Additional regions of cortical thinning in the left rostral middle frontal gyrus, left caudal middle frontal gyrus and right superior frontal gyrus were identified when PSP were compared with PD (Figure 1, Table 2). There were no significant regions of cortical thinning in the PD, MSA or HC groups.

**Table 2:** Cortical regions displaying thinning of the cortex and increased surface area in PSP.

Contrast	Region	Coordinates	Vertex	Value	Size
		X, Y, Z			(mm^2^)
**Thickness**					
PSP—HC	Left precentral gyrus	−5.88, 2.49, 57.62	0	−4.00	2212.57
	Left superior frontal gyrus	30.06, 16.56, 43.15	2	−2.85	1229.26
	Right superior frontal gyrus	−31.80, 60.96, 21.96	4	−4.00	5516.95
	Right lateral orbitofrontal gyrus	−20.67, 56.64, −52.51	9	−3.00	1236.09
PSP—MSA	Left superior frontal gyrus	10.05, 30.67, 59.18	51	−3.10	1283.04
	Right superior frontal gyrus	−31.80, 60.96, 21.96	4	−4.00	3166.28
PSP—PD	Left rostral middle frontal gyrus	−1.20, 98.31, −6.90	55	−3.52	1372.08
	Left caudal middle frontal gyrus	−23.55, 39.37, 22.68	94	−3.10	1317.47
	Right superior frontal gyrus	−31.80, 60.96, 21.96	4	−4.00	3043.13
**Surface Area**					
PSP—MSA	Left pericalcarine	24.95, −100.10, −20.18	34	4.00	1864.52

X;Y;Z in Talairach coordinates. All results presented at the corrected threshold (p <0.05).

MSA  =  Multiple Systems Atrophy; PSP  =  Progressive Supranuclear Palsy; PD  =  Parkinson's Disease.

### Surface area

**Figure 2:**
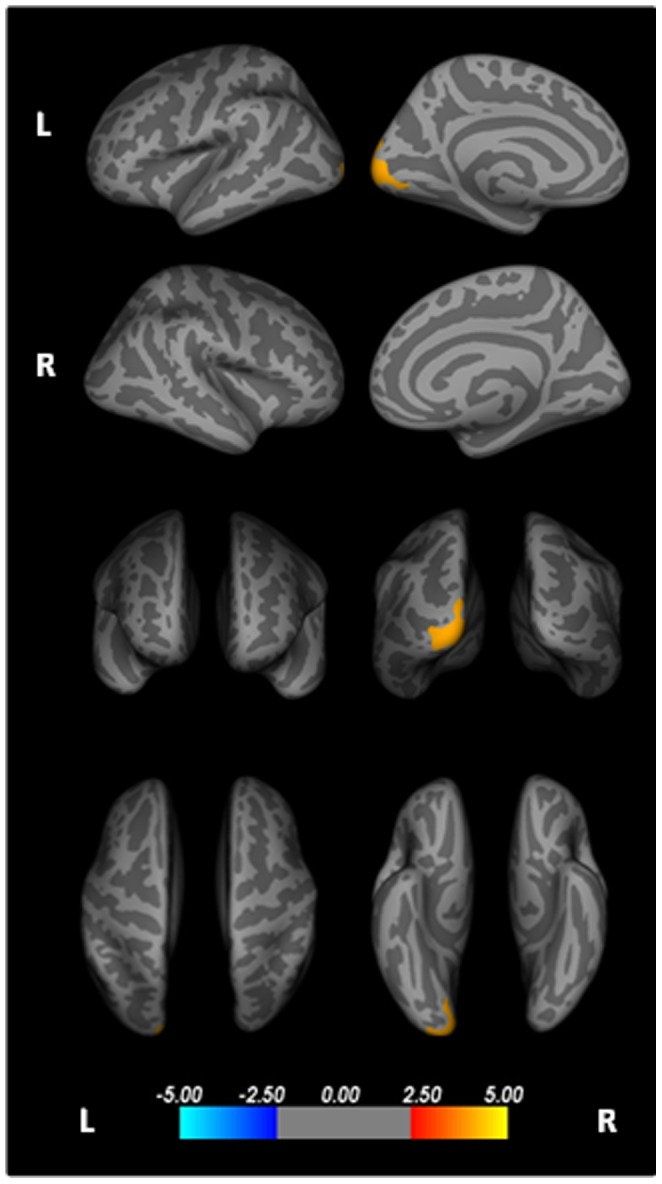
Cortical areas showing significantly increased surface area in PSP patients compared to Multiple System Atrophy; displayed on QDEC's semi-inflated cortical surfaces. Top row: L lateral and L medial, Second row: R lateral and R medial, Third row: anterior and posterior, Bottom row: superior and inferior views. The colour bar indicates the significance levels of the clusters. Results were obtained using Monte Carlo simulation, with a threshold of p <0.05, to provide cluster-wise correction for multiple comparisons.

PSP patients displayed significantly increased surface area in the left pericalcarine when compared with the MSA group (Figure 2, Table 2). No regions of significantly increased or decreased surface area were detected in PD, MSA or HC groups.

### Cortical volume

Cortical volume loss was detected in the superior frontal gyrus bilaterally in the PSP group when compared with controls (Figure S1) and the right superior frontal gyrus when compared with PD (Figure S1, Table S1). No significant differences in volume were detected in the PD, MSA or HC groups.

### Correlation between cortical and clinical measures

Spearman correlation analyses showed no significant correlation between any clinical or cognitive measures with thickness, surface area or volume in any of the subject groups.

### Effect of cognitive impairment

Finally, when patients with DRS scores below the threshold of cognitive impairment (DRS score ≤ 125) were excluded from the between group analysis, results were very similar to the initial group comparisons, with widespread frontal thinning remaining a significant result (Table S2).

## Discussion

To our knowledge this is the first study to quantify and compare surface-based measures in PD, MSA and PSP. Our results show that patients with PSP display consistent thinning and volume loss of the superior frontal gyrus, as well as thinning of the left precentral, rostral middle frontal, caudal middle frontal and right lateral orbitofrontal gyri. Additionally, the left pericalcarine displayed a significant increase in surface area in the PSP group. There were no significant results in the MSA or PD groups. These findings support the hypothesis that PSP patients display more clinically severe symptoms as well as differential and more established patterns of cortical atrophy that particularly affects the frontal lobe.

The topographic pattern of cortical thinning and volume loss in the superior frontal region seen here, is similar to that found in recent studies,[8],[30] reflecting that the superior regions of the frontal lobe may be particularly targeted in PSP. Previous functional imaging studies have also found abnormalities in this region with reduced glucose metabolism in the superior half of the frontal lobes [31] and reduced regional cerebral perfusion has been reported in a SPECT study.[32] It may be interesting to highlight that the frontal lobe receives projections from white matter tracts such as the superior cerebellar peduncle and superior longitudinal fasciculus. While the results from this study show grey matter thinning of frontal regions bilaterally, a loss of integrity of the superior cerebellar peduncles has also been found in separate studies.[33],[34] Furthermore, previous studies have shown deep grey matter structure degeneration [30] and loss of integrity of various other white matter fibres [30],[34] - it is possible that in degenerative diseases such as PSP, the build-up of subcortical tau deposition is followed by white matter tract degeneration [30],[33] which subsequently causes neuronal loss, reflected by thinning of the cortex. It would be beneficial for future studies to combine surface-based measures with DTI measures to fully assess the relationship between the cerebral cortex and white matter connectivity.

Results from this study also showed significantly increased surface area in the pericalcarine cortex of the occipital lobe in the PSP group. It is noteworthy that reduced fractional anisotropy (FA) reflecting loss of integrity has previously been identified in the white matter of the pericalcarine in a population of PD patients; this result was further correlated with poor cognitive flexibility. PSP patients often display cognitive impairments similar to those seen in PD, although often in a markedly more severe form [29],[35] (Table 1). Although the measures from the pericalcarine did not correlate with cognitive measures in this study, when cognitively impaired patients were excluded from the analysis, a significant increase in surface area could no longer be seen in this region, therefore it is likely that this result was driven by those cognitively impaired patients and thus related to cognitive impairment.

Surprisingly, no changes were detected in the PD group after Monte Carlo Simulation correction for multiple comparisons was applied. Previous studies have reported inconsistent results in the pathology of PD, with some studies reporting involvement of the frontal, occipital, parietal and temporal regions of the cortex,[15],[36] whilst others report no significant differences.[7] Of these studies finding significant atrophy in PD only Pereira and colleagues [15] were able to identify regions of cortical thinning that were robust enough to survive Monte Carlo Simulation. Considering this study made use of the same FreeSurfer analysis pipeline, although an earlier version (version 4.3.1) and the group mean age (64 years) was very close to that seen in the present study, it is likely that the more robust findings seen in their study is due to the use of a 3T scanner as opposed to a 1.5T scanner as used in the present study. 3T scanners allow a higher signal-to-noise ratio (SNR) and increased spatial resolution; these improvements in terms of image quality may allow more subtle differences in cortical morphometry to be detected at the analysis stage.

To our knowledge this is the first study to assess cortical thickness and surface area in an MSA population. Our results show that there are no significant regions of cortical change in MSA patients, however a previous VBM study has identified frontal and motor areas of volume loss in an MSA sample [7] – thus this patient group needs further investigation with a larger sample size to increase the statistical power. In addition, it is plausible that patients with MSA-P and MSA-C display differential patterns of cortical atrophy in association with the different symptoms shown in the MSA variants. Thus, it would be beneficial for future studies to assess these two groups separately and compare the results with differential patterns of symptoms seen between them.

Overall, these results confirm that surface-based methods are a sensitive tool with which to measure disease related changes of the cerebral cortex. In fact, cortical thinning was observed in regions of the frontal lobe in the PSP group, where neither volume loss nor surface area changes were detected. While there was no significant change in surface area in the superior frontal gyrus it is plausible to reason that the volume loss displayed here and potentially in previous volumetric studies is driven by cortical thinning alone. These findings add support to the concept that these morphometric measures are completely separate and may reflect different pathological changes, thus emphasising the importance of assessing cortical thickness and surface area as separate measures and reiterate the focused sensitivity of surface-based methods.

The present study is advantageous over previous similar studies because it includes data from three clinical groups included in a single study, however the statistical power of the study would be increased with larger group sizes. In future it would therefore be beneficial to recruit a larger sample of participants from each clinical group. In addition, results may have been affected by the slightly older age of the PSP patients, thus age was included as a nuisance factor in all analyses to account for this difference. A final limitation of the study is that the spatial resolution of the data used was not isotropic, thus there is a potential possibility that subtle changes in the cortex may have gone unnoticed.

In conclusion, this study has identified widespread frontal atrophy in PSP, in particular the superior frontal gyrus, enabling morphological differentiation of this disease from PD and MSA at the clinically established disease stage. It may be interesting in the future to extend these results by using similar methods to those that have previously been employed in these patient groups [37],[38], this would enable us to determine whether these patient groups could be correctly classified based on the cortical atrophy seen in the current study, thus establishing the potential for use in clinical practice.

## Supporting Information

Figure S1 Cortical areas showing significant volume loss in PSP patients compared to a) healthy controls, b) Parkinson's disease; displayed on QDEC's semi-inflated cortical surfaces. Top row: L lateral and L medial, Second row: R lateral and R medial, Third row: anterior and posterior, Bottom row: superior and inferior views. The colour bar indicates the significance levels of the clusters. Results were obtained using Monte Carlo simulation, with a threshold of p <0.05, to provide cluster-wise correction for multiple comparisons.
(TIF)

Table S1 Cortical regions displaying volume loss in PSP. X;Y;Z in Talairach coordinates. All results presented at the corrected threshold (p <0.05).
(DOCX)

Table S2 Cortical regions displaying thinning of the cortex and increased surface area in PSP, excluding patients with a DRS score ≤ 125. X;Y;Z in Talairach coordinates. All results presented at the corrected threshold (p <0.05).
(DOCX)
