# Supplementary figures and images for: Cortical Thickness, Surface Area and Volume Measures in Parkinson's Disease, Multiple System Atrophy and Progressive Supranuclear Palsy
Source: PLoS One. 2014 Dec 2;9(12):e114167. doi: 10.1371/journal.pone.0114167 (PMC4252086; doi:10.1371/journal.pone.0114167)

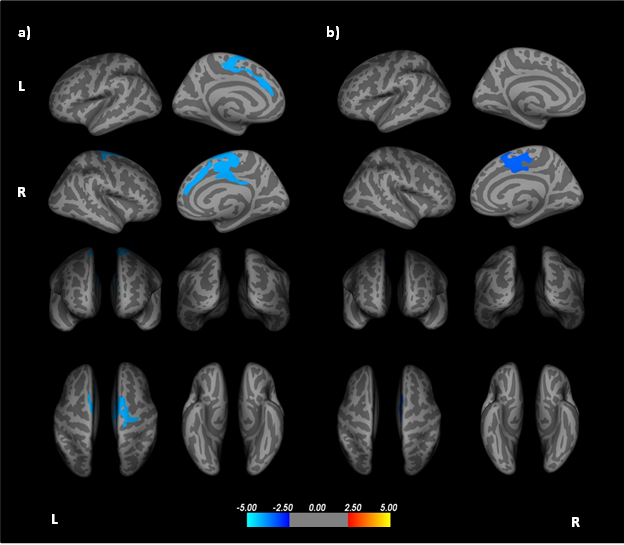

Supplement: Figure S1 — Cortical areas showing significant volume loss in PSP patients compared to a) healthy controls, b) Parkinson's disease; displayed on QDEC's semi-inflated cortical surfaces. Top row: L lateral and L medial, Second row: R lateral and R medial, Third row: anterior and posterior, Bottom row: superior and inferior views. The colour bar indicates the significance levels of the clusters. Results were obtained using Monte Carlo simulation, with a threshold of p <0.05, to provide cluster-wise correction for multiple comparisons. (TIF) [file pone.0114167.s001.tif]
