# Supplementary material for: Cortical Thickness, Surface Area and Volume Measures in Parkinson's Disease, Multiple System Atrophy and Progressive Supranuclear Palsy
Source: PLoS One. 2014 Dec 2;9(12):e114167. doi: 10.1371/journal.pone.0114167 (PMC4252086; doi:10.1371/journal.pone.0114167)
Supplement: Table S1 — Cortical regions displaying volume loss in PSP. X;Y;Z in Talairach coordinates. All results presented at the corrected threshold (p <0.05). (DOCX) [file pone.0114167.s002.docx]

| Contrast | Region | Coordinates | Vertex | Value | Size |
| --- | --- | --- | --- | --- | --- |
|  |  | X, Y, Z |  |  | (mm^2^) |
| **Volume** |  |  |  |  |  |
| PSP—HC | Left superior frontal gyrus | 30.06, 16.56, 43.15 | 2 | -4.00 | 1357.05 |
|  | Right superior frontal gyrus | -31.80, 60.96, 21.96 | 4 | -4.00 | 3573.49 |
| PSP—PD | Right superior frontal gyrus | -30.66, 24.39, 50.08 | 100 | -3.15 | 1186.83 |
| MSA = Multiple Systems Atrophy; PSP = Progressive Supranuclear Palsy; PD = Parkinson's Disease. | | | | | |
