# Supplementary material for: Cortical Thickness, Surface Area and Volume Measures in Parkinson's Disease, Multiple System Atrophy and Progressive Supranuclear Palsy
Source: PLoS One. 2014 Dec 2;9(12):e114167. doi: 10.1371/journal.pone.0114167 (PMC4252086; doi:10.1371/journal.pone.0114167)
Supplement: Table S2 — Cortical regions displaying thinning of the cortex and increased surface area in PSP, excluding patients with a DRS score ≤ 125. X;Y;Z in Talairach coordinates. All results presented at the corrected threshold (p <0.05). (DOCX) [file pone.0114167.s003.docx]

| Contrast | Region | Coordinates | Vertex | Value | Size |
| --- | --- | --- | --- | --- | --- |
|  |  | X, Y, Z |  |  | (mm^2^) |
| **Thickness** |  |  |  |  |  |
| PSP—HC | Left precentral gyrus | -5.88, 2.49, 57.62 | 0 | -4.00 | 2163.41 |
|  | Left rostral middle frontal gyrus | -8.84, 78.89, 13.67 | 57 | -2.82 | 1224.31 |
|  | Right superior frontal gyrus | -31.80, 60.96, 21.96 | 4 | -4.00 | 4841.50 |
| PSP—MSA | Left superior frontal gyrus | 10.05, 30.67, 59.18 | 51 | -2.52 | 1114.68 |
|  | Right paracentral lobule | -30.3, -7.84, 52.65 | 99 | -2.21 | 1026.96 |
| PSP—PD | Left precentral gyrus | -27.39, 19.4, 30.24 | 12 | -4 | 4140.13 |
|  | Left superior frontal gyrus | -30.06, 16.56, 43.15 | 2 | -2.2 | 1008.66 |
|  | Right superior frontal gyrus | -31.80, 60.96, 21.96 | 4 | -4.00 | 2555.5 |
| **Surface Area** |  |  |  |  |  |
| PSP—MSA | Right pars triangularis | 21.85, 63.78, -16.43 | 22 | 4 | 1318.63 |
| **Volume** |  |  |  |  |  |
| PSP—HC | Left superior frontal gyrus | 29.87, 14.84, 58.47 | 105 | -2.62 | 1003.77 |
|  | Right superior frontal gyrus | -31.80, 60.96, 21.96 | 4 | -4.00 | 2229.78 |
| PSP—PD | Right posterior cingulate gyrus | -32.23, 33.8, 32.71 | 62 | -3.10 | 1149 |
| MSA = Multiple Systems Atrophy; PSP = Progressive Supranuclear Palsy; PD = Parkinson's Disease. | | | | | |
